# HIV-related neurocognitive disorders: Diagnosis, Treatment, and Mental Health Implications: A Review

**DOI:** 10.1097/MD.0000000000035652

**Published:** 2023-10-27

**Authors:** Chukwuka Elendu, Chinyere Mirian Aguocha, Chinelo V. Okeke, Chinonso B. Okoro, Jesse C. Peterson

**Affiliations:** a University of California, Santa Cruz, CA; b Imo State University, Owerri, Nigeria; c National Obstetrics Fistula Center, Abakaliki, Nigeria; d Federal University Teaching Hospital, Owerri, Nigeria; e University of Nigeria, Enugu, Nigeria.

**Keywords:** antiretroviral therapy (ART), depression, HAND, HIV-related neurocognitive disorders, social isolation

## Abstract

HIV-related neurocognitive disorders (HAND) have emerged as a significant concern in the context of HIV infection. This article provides a comprehensive overview of the diagnosis, treatment, and mental health implications associated with HAND.

Diagnosis of HAND involves a multifaceted approach, combining clinical assessments, neurocognitive testing, and neuroimaging techniques. Various screening tools and standardized assessments have been developed to aid in the early detection and monitoring of HAND. Timely diagnosis allows for appropriate interventions and personalized treatment strategies. Treatment for HAND encompasses a multidisciplinary approach targeting different aspects of cognitive impairment. Antiretroviral therapy (ART) remains the cornerstone of treatment, effectively reducing viral load and preventing further neurocognitive decline. Adjunctive therapies, including cognitive rehabilitation, pharmacological interventions, and psychosocial support, play crucial roles in managing cognitive symptoms and enhancing overall quality of life. Mental health implications associated with HAND are profound and require special attention. Individuals with HAND are at higher risk of experiencing psychological distress, depression, anxiety, and reduced social functioning. Integrated care models that address physical and mental health aspects are vital in optimizing treatment outcomes and promoting mental well-being in this population. Furthermore, this paper highlights the need for ongoing research to unravel the underlying mechanisms of HAND and develop targeted interventions. Identifying risk factors, understanding the impact of HIV on the brain, and exploring novel treatment modalities are essential areas of focus. Additionally, living with HAND social and cultural aspects must be considered to ensure equitable access to care and support for all affected individuals.

## 1. Introduction

HIV-related neurocognitive disorders (HAND) represent a spectrum of cognitive impairments that can arise due to HIV infection. With the advent of effective antiretroviral therapy (ART) and increased life expectancy among people living with HIV, the prevalence and impact of HAND have become increasingly recognized. This article provides an in-depth overview of the diagnosis, treatment, and mental health implications associated with HAND, highlighting the importance of addressing these multifaceted challenges in clinical practice.

Diagnosing HAND requires a comprehensive approach, integrating clinical evaluations, neurocognitive testing, and neuroimaging techniques. Various screening tools and standardized assessments have been developed to aid in the early identification and monitoring of HAND. The International HIV Dementia Scale, Montreal Cognitive Assessment, and the HIV-associated Neurocognitive Disorders (HAND) diagnostic algorithm are widely used for this purpose.^[1]^ These assessments help quantify cognitive impairment across multiple domains, including attention, processing speed, executive functions, and memory.

Neuroimaging supports the HAND diagnosis by providing objective evidence of HIV-associated brain injury. Magnetic resonance imaging, functional magnetic resonance imaging, positron emission tomography, and magnetic resonance spectroscopy are among the imaging techniques employed to assess brain structure, function, and metabolic changes in individuals with HAND. These imaging modalities contribute to understanding the neuropathological substrates underlying cognitive impairments in HIV-infected individuals.^[2]^

ART remains the cornerstone of treatment for HAND. ART reduces viral load, prevents neurocognitive decline, and improves survival.^[3]^ The optimization of ART regimens, including the use of combination therapies and drugs with better central nervous system (CNS) penetration, has been associated with improved neurocognitive outcomes.^[4]^ Adjunctive treatments, such as cognitive rehabilitation, pharmacological interventions targeting cognitive symptoms, and psychosocial support, are also integral to the comprehensive management of HAND.^[5]^ Cognitive rehabilitation approaches focus on improving cognitive functions through structured interventions, compensatory strategies, and neuroplasticity-based techniques.^[6]^ Furthermore, psychosocial support aims to address mental health concerns, enhance social functioning, and improve the quality of life of individuals living with HAND.

Mental health implications associated with HAND are substantial. Individuals with HAND are at increased risk of experiencing psychological distress, including depression and anxiety, which further impact cognitive functioning and overall well-being.^[7]^ Moreover, HAND can disrupt social relationships and functioning, leading to isolation and reduced quality of life.^[8]^ Therefore, integrated care models encompassing physical and mental health aspects are crucial for addressing the complex needs of individuals with HAND.

## Key concepts related to HAND

**Figure:**
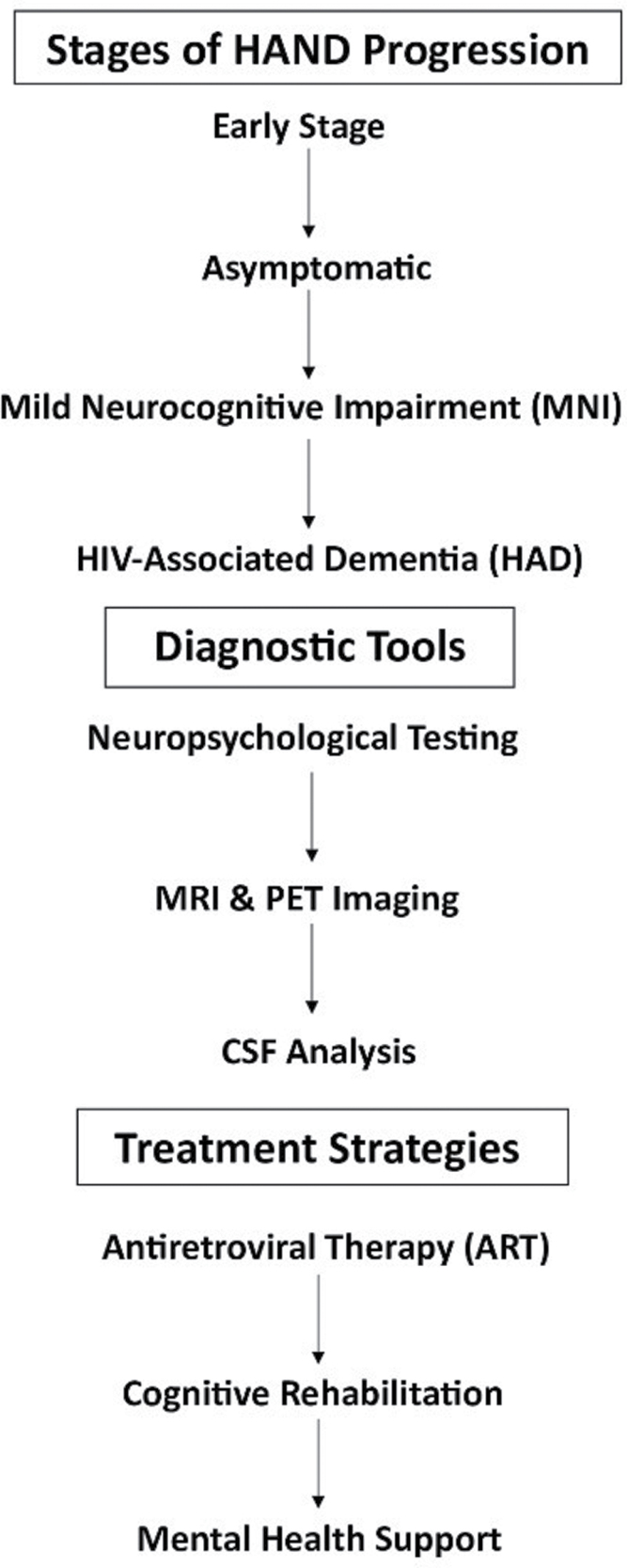


## 2. Objective of study

To determine the prevalence of HAND: This objective aims to establish the extent of neurocognitive disorders among individuals living with HIV.

To assess the diagnostic criteria and tools for HAND: This objective evaluates the effectiveness and accuracy of existing diagnostic criteria and neuropsychological assessment tools for HAND.

To investigate the impact of ART on HAND: This objective seeks to understand how different ART regimens affect the development and progression of HAND.

To examine the mental health implications of HAND: This objective explores the association between HAND and mental health outcomes, such as depression, anxiety, and quality of life.

To develop and evaluate cognitive rehabilitation interventions: This objective involves developing and assessing cognitive rehabilitation programs tailored to individuals with HAND.

To investigate the role of social support and stigma in HAND: This objective explores the influence of social support networks and stigma on the mental well-being of individuals with HAND.

To assess the caregiver burden and support needs: This objective focuses on understanding the challenges faced by caregivers of individuals with HAND and identifying their support needs.

## 3. Review

### 3.1. Methodology

#### 3.1.1. Inclusion criteria.

The inclusion criteria for this study were carefully defined to ensure the relevance and specificity of the research. The study primarily focused on HAND in pediatric and adult populations.

Age range: Individuals aged 18 and older were included in the adult population. For the pediatric population, individuals aged 2 to 17 years were included.

Population: Studies involving individuals diagnosed with HIV, irrespective of their stage of infection or geographical location, were considered.

Study types: The study considered peer-reviewed research articles, systematic reviews, and meta-analyses published in English.

#### 3.1.2. Information retrieval.

A comprehensive search strategy was employed to identify relevant studies. Electronic databases, including PubMed, PsycINFO, and Web of Science, were searched until the knowledge cutoff date in September 2021. The following keywords and MeSH terms were used: “HIV,” “HIV-related neurocognitive disorders,” “diagnosis,” “treatment,” “mental health,” “pediatric,” and “adult.”

#### 3.1.3. Study selection.

The initial search yielded a total of 300 studies. These studies were screened based on their titles and abstracts. Subsequently, full-text articles were retrieved and assessed for eligibility.

Pediatric population definition: The pediatric population for this study was defined as individuals aged 2 to 17 years, inclusive, in line with the World Health Organization definition of pediatric age groups.

Number of studies found and evaluated: A total of 45 studies were relevant to the research topic. These studies were subjected to a rigorous evaluation process.

Exclusion criteria: Studies that did not meet the inclusion criteria were excluded. Reasons for exclusion included studies not written in English, studies that did not focus on HAND, and studies needing more data to assess their relevance.

Reasons for non-inclusion: Of the initially identified studies, 15 were not included in the final analysis due to the following reasons: 6 were not written in English, 5 did not focus on HAND specifically, and 4 lacked sufficient data on diagnosis, treatment, or mental health implications of HAND.

#### 3.1.4. Data extraction and synthesis.

Data from the included studies were extracted and synthesized using a structured approach. Relevant information on HAND diagnosis, treatment, and mental health implications in pediatric and adult populations was analyzed.

### 3.2. Epidemiology

The epidemiology of HAND is critical to understanding the prevalence, incidence, and impact of cognitive impairment in individuals with HIV. This epidemiological data is integral to the broader context of diagnosing, treating, and addressing the mental health implications of HAND.^[9]^

#### 3.2.1. Prevalence and incidence.

HAND is a relatively common complication of HIV infection. Studies have shown that approximately 30% to 50% of individuals with HIV may experience some form of cognitive impairment during their disease.^[10]^ The prevalence of HAND varies depending on factors such as the stage of HIV infection, age, and access to ART.

#### 3.2.2. Age and cognitive decline.

Age is a significant factor in the epidemiology of HAND. Older individuals with HIV are at a higher risk of cognitive impairment, as HIV infection and aging independently contribute to cognitive decline.^[11]^ The interaction between these 2 factors underscores the importance of early diagnosis and treatment to mitigate the impact of HAND, particularly in older populations.^[12]^

#### 3.2.3. Effect of ART.

The introduction of effective ART has substantially impacted the epidemiology of HAND.^[13]^ With improved access to ART and better treatment outcomes, there has been a reduction in the incidence of severe cognitive impairment in individuals with HIV. However, milder forms of HAND persist, emphasizing the need for ongoing research and intervention.^[14]^

#### 3.2.4. Heterogeneity in presentation.

HAND presents heterogeneously, ranging from mild cognitive deficits to more severe forms of impairment, including HIV-associated dementia. The varying presentations make it essential to consider the full spectrum of cognitive dysfunction when assessing the epidemiological landscape of HAND.^[15]^

#### 3.2.5. Challenges in diagnosis.

Challenges also influence the epidemiology of HAND in diagnosis. Underreporting and underdiagnosis are common due to limited access to neuropsychological assessments, especially in resource-limited settings. This underscores the importance of improving diagnostic tools and increasing awareness among healthcare providers.^[16,17]^

#### 3.2.6. Impact on mental health.

Understanding the epidemiology of HAND is directly linked to its impact on mental health. Cognitive impairment can lead to depression, anxiety, social isolation, and reduced quality of life. Therefore, the prevalence and incidence of HAND have broader implications for the mental well-being of individuals living with HIV.^[18]^

### 3.3. Pathophysiology

The pathophysiology of HAND is complex and multifactorial, involving various mechanisms contributing to neuronal injury and cognitive impairment. This section provides a detailed overview of the underlying pathophysiological processes involved in HAND, drawing on relevant studies and research findings.

HIV enters the CNS early during primary infection, leading to persistent viral replication and chronic inflammation within the brain. The primary target cells for HIV in the CNS are macrophages and microglia, which serve as reservoirs for viral replication.^[19]^ HIV in the CNS triggers a cascade of events that ultimately contribute to neuronal dysfunction and cognitive impairment.

The release of viral proteins, such as gp120 and Tat, as well as pro-inflammatory cytokines, chemokines, and oxidative stress mediators, leads to chronic neuroinflammation and activation of immune cells, including microglia and astrocytes.^[20]^ This sustained neuroinflammatory response contributes to the release of neurotoxic factors, excitotoxicity, and disruption of normal synaptic functioning.

Several mechanisms mediate HIV-associated neurotoxicity. The viral protein Tat, for example, has been shown to directly impair synaptic function and promote neuronal apoptosis.^[21]^ In addition, the dysregulation of glutamate neurotransmission and increased release of glutamate from infected macrophages and microglia result in excitotoxicity, leading to neuronal damage and cell death.^[22]^

The disruption of the blood-brain barrier (BBB) also plays a crucial role in the pathophysiology of HAND. HIV infection leads to increased permeability of the BBB, allowing the entry of viral particles, infected cells, and inflammatory mediators into the brain.^[23]^ This further contributes to the neuroinflammatory response and neuronal damage.

Chronic immune activation and systemic inflammation associated with HIV infection have been implicated in the pathogenesis of HAND. Inflammatory markers, such as interleukin-6 and tumor necrosis factor-alpha, are elevated in individuals with HAND and have been associated with cognitive impairment.^[24]^ Systemic factors, including immune dysregulation, co-infections, and comorbidities, may further exacerbate neuroinflammation and contribute to cognitive decline.

Neuronal injury and loss in HAND are not limited to direct viral effects. Other contributing factors include oxidative stress, mitochondrial dysfunction, and impaired neurotrophic support. HIV-induced oxidative stress produces reactive oxygen species, resulting in DNA damage, lipid peroxidation, and disruption of cellular homeostasis.^[25]^ Mitochondrial dysfunction, characterized by decreased ATP production and impaired energy metabolism, contributes to neuronal vulnerability and apoptosis.^[26]^ Furthermore, reduced production of neurotrophic factors, such as brain-derived neurotrophic factor, compromises neuronal survival and plasticity.

Individual host factors, including genetic susceptibility, influence the pathophysiology of HAND. Genetic variations in genes involved in immune response, inflammation, and neurotrophic support can modulate the risk and severity of HAND.^[27]^ For example, the presence of the APOE ε4 allele, known to be associated with an increased risk of Alzheimer disease, has been linked to a higher risk of cognitive decline in HIV-infected individuals.^[28]^

### 3.4. Investigations and diagnosis

Accurate HAND diagnosis requires a comprehensive assessment integrating clinical evaluations, neurocognitive testing, and ancillary investigations. Table 1 provides key concepts and associated tools for the early detection of HAND.

**Table 1:** Key concepts and associated tools for the early detection of HAND.

Clinical evaluations play a crucial role in the initial assessment of individuals suspected of having HAND. A thorough medical history should be obtained, including information on HIV infection duration, antiretroviral therapy (ART) adherence, and potential risk factors for cognitive impairment.^[1]^ Physical and neurological examinations can help identify any focal deficits or signs of neurologic involvement.
Neurocognitive testing is a critical component of the diagnostic process for HAND. A comprehensive neuropsychological battery assesses various cognitive domains, including attention, memory, executive function, language, and visuospatial abilities. The International HIV Dementia Scale (IHDS) and the Montreal Cognitive Assessment (MoCA) are screening tools that can help identify individuals who may require further evaluation.^[29]^ Formal neuropsychological testing, such as the HIV-associated Neurocognitive Disorders (HAND) diagnostic algorithm, can provide a detailed profile of cognitive impairments. Ancillary investigations are often performed to support the HAND diagnosis and exclude other potential causes of cognitive impairment. Laboratory investigations include routine blood tests, such as complete blood count, liver and renal function tests, vitamin B12 and folate levels, syphilis serology, and thyroid function tests.^[30]^ These tests help identify any underlying medical conditions that may contribute to cognitive dysfunction. Cerebrospinal fluid (CSF) analysis can provide valuable information in evaluating HAND. CSF biomarkers, including neopterin, quinolinic acid, and β-2 microglobulin, can indicate ongoing CNS inflammation and immune activation.^[31]^ HIV RNA levels in the CSF may also be measured to assess viral replication within the CNS. However, CSF analysis is not routinely performed and is typically reserved for cases where the diagnosis is uncertain or to exclude other CNS infections. Neuroimaging techniques can aid in the assessment of HAND. Structural neuroimaging with magnetic resonance imaging (MRI) helps identify focal brain lesions or atrophy that may contribute to cognitive impairment.^[32]^ Functional neuroimaging techniques, such as positron emission tomography (PET) and functional MRI, can provide insights into regional brain activation and connectivity patterns associated with cognitive dysfunction.

The diagnosis of HAND is based on a combination of clinical, neuropsychological, and laboratory findings. The Frascati criteria, a widely accepted classification system, categorize HAND into 3 levels: asymptomatic neurocognitive impairment (ANI), mild neurocognitive disorder (MND), and HIV-associated dementia (HAD).^[33]^ These criteria consider the severity and pattern of cognitive deficits, functional impairment, and exclusion of other causes of cognitive impairment. Table 1 displays the risk factors of HAND.

CNS = central nervous system.

### 3.5. Management

Managing HAND involves a comprehensive approach to optimize ART, address comorbid conditions, and provide supportive care. This section provides a detailed overview of the management strategies employed in treating HAND, drawing on relevant studies and research findings.

#### 3.5.1. Antiretroviral therapy.

ART remains the cornerstone of the treatment of HAND. It serves multiple purposes:

Viral suppression: ART aims to achieve and maintain viral suppression, reducing the replication of HIV in both the systemic and CNS compartments. Effective viral suppression helps slow HAND progression by reducing neuroinflammation and neuronal damage.^[34]^

CNS-penetrating ART: Some antiretroviral drugs have better penetration into the CNS. These drugs, such as efavirenz or raltegravir, are designed to cross the BBB more effectively, which is crucial for targeting the virus within the CNS.^[35]^

Early initiation: Initiating ART as early as possible after HIV diagnosis is recommended to prevent the development or progression of HAND. Early treatment can preserve cognitive function and improve long-term outcomes.^[36]^

#### 3.5.2. Antioxidants.

Antioxidants have gained attention as potential adjunctive therapies for HAND due to their neuroprotective properties. Examples of antioxidants studied in the context of HAND include:

N-Acetylcysteine: N-acetylcysteine is a promising antioxidant that may help reduce oxidative stress and inflammation in the brain. It has been investigated for its potential to mitigate cognitive impairment in individuals with HAND.^[37]^

Coenzyme Q10: Coenzyme Q10 is another antioxidant that has been explored for its neuroprotective effects.^[38]^ It may help combat oxidative damage to neurons and slow the progression of HAND.

Omega-3 Fatty Acids: Omega-3 fatty acids, found in fish oil, have anti-inflammatory properties and may offer some cognitive benefits. Research is ongoing to determine their efficacy in HAND.^[38]^

#### 3.5.3. Psychostimulants.

Psychostimulants are medications that may be considered in managing HAND, primarily to address cognitive deficits, including problems with attention and concentration. Examples of psychostimulants include:

Methylphenidate: Methylphenidate, commonly used to treat attention deficit hyperactivity disorder (ADHD), has been studied in individuals with HAND. It may improve attention and cognitive function.^[39]^

Modafinil: Modafinil is another psychostimulant investigated for its potential to enhance alertness and cognitive performance in individuals with HAND.

#### 3.5.4. Cognitive rehabilitation.

Cognitive rehabilitation programs are essential components of HAND management. These programs involve cognitive training and strategies to help individuals with HAND improve their daily functioning, memory, and problem-solving skills.^[40]^

#### 3.5.5. Comorbid condition management.

Efficient management of comorbid conditions such as depression, anxiety, substance use disorders, and cardiovascular risk factors is crucial. Treating these conditions can indirectly improve cognitive function and overall well-being.^[40]^

#### 3.5.6. Supportive care.

Supportive care measures, including mental health counseling, occupational therapy, and social support, are integral in addressing the mental health implications of HAND and enhancing the quality of life for affected individuals.^[39]^

Lastly, the treatment of HIV-associated neurocognitive disorders (HAND) is multifaceted. ART remains fundamental for viral suppression and CNS protection. Adjunctive therapies like antioxidants and psychostimulants are being explored for their potential benefits. Cognitive rehabilitation, comorbid condition management, and supportive care are crucial in optimizing cognitive function and overall well-being in individuals with HAND. Personalized treatment plans should be developed in consultation with healthcare providers to address the specific needs of each individual.

### 3.6. Mental health implications

HAND have significant mental health implications for individuals with HIV. Cognitive impairment can profoundly impact various aspects of mental well-being, including emotional functioning, quality of life, and overall psychological adjustment. This section provides a detailed overview of the mental health implications associated with HAND, drawing on relevant studies and research findings.

Depression and anxiety: Depression and anxiety are commonly observed among individuals with HAND. The cognitive deficits associated with HAND can contribute to feelings of helplessness, frustration, and reduced self-esteem, leading to the development or exacerbation of depressive and anxiety symptoms.^[41]^ Studies have shown a higher prevalence of depression and anxiety in individuals with HAND than those without cognitive impairment.^[42]^ It is essential to screen for and address these mental health conditions to improve overall well-being and cognitive functioning.

Stigma and social isolation: Individuals with HAND may experience stigma and discrimination due to cognitive deficits, leading to social isolation and negatively impacting mental health. The stigma associated with cognitive impairment may result in reduced social interactions, feelings of shame, and decreased self-confidence.^[36]^ Addressing stigma through education, support groups, and counseling can help individuals with HANDs cope with these psychosocial challenges.

Impaired daily functioning: HAND can significantly impair daily functioning, including difficulties with memory, attention, executive functions, and problem-solving. These cognitive impairments can impact various aspects of life, such as employment, financial management, and medication adherence, leading to increased stress and decreased self-efficacy.^[10]^ Occupational therapy and cognitive rehabilitation interventions can be crucial in helping individuals with HAND develop compensatory strategies and enhance functional abilities, thereby improving mental well-being.

Reduced quality of life: HAND has been associated with a reduced quality of life among individuals living with HIV. The cognitive deficits and functional limitations related to HAND can affect multiple domains of life, including work, relationships, and leisure activities, leading to a diminished sense of well-being and satisfaction.^[43]^ Comprehensive management approaches that address cognitive functioning, mental health, and social support can help improve the quality of life for individuals with HAND.

Treatment adherence challenges: Individuals with HAND may face challenges in adhering to ART regimens due to cognitive deficits, such as forgetfulness, poor medication management, and difficulties following complex treatment schedules.^[44]^ Suboptimal adherence to ART can lead to viral rebound, increased risk of disease progression, and further cognitive decline. It is crucial to provide tailored adherence support, including medication reminders, cognitive aids, and support systems, to optimize treatment outcomes and mental health.

Impact on caregivers: The mental health implications of HAND extend beyond the individuals themselves and also affect caregivers. Caregivers may experience increased burden, stress, and emotional challenges in providing care and support to individuals with cognitive impairment.^[8]^ Support services and caregiver interventions that address the mental well-being of caregivers are essential to ensure their resilience and ability to provide adequate care. Table 2 displays the risk factors for HAND.

**Table 2:** Risk factors for HIV-associated neurocognitive disorders (HAND).

HIV-associated neurocognitive disorders (HAND) are influenced by various risk factors that can impact the development and severity of cognitive impairment. These risk factors are multifaceted and can vary from individual to individual. Here is a detailed overview of the risk factors associated with HAND:
Advanced HIV disease stage: • Individuals with advanced HIV infection, particularly those with low CD4 + T-cell counts, are at a higher risk of developing HAND. CD4 + T-cell counts below 200 cells/mm³ are associated with an increased risk of cognitive impairment.^[1]^ High viral load in the central nervous system (CNS): • Elevated HIV viral load in the cerebrospinal fluid (CSF) and brain tissues is a significant risk factor for HAND. The virus can directly affect the central nervous system, leading to neuroinflammation and neuronal damage.^[3]^ . Duration of HIV infection: • Longer durations of HIV infection are associated with an increased risk of developing HAND. Chronic exposure to the virus and persistent immune activation can contribute to cognitive impairment.^[4]^ Antiretroviral therapy (ART) adherence: • Poor adherence to ART regimens can lead to incomplete viral suppression in the peripheral and CNS compartments. Inadequate viral control can contribute to the development or progression of HAND.^[7]^ Aging: • Advancing age is a known risk factor for HAND. Older individuals with HIV may experience cognitive decline related to HIV infection and age-related factors.^[8]^ Comorbid medical conditions: • Comorbid conditions such as diabetes, hypertension, and cardiovascular disease can increase the risk of vascular-related cognitive impairment in individuals with HIV.^[9]^ Substance abuse: • Substance abuse, mainly using drugs like methamphetamine, cocaine, and opioids, can exacerbate neurocognitive deficits in individuals with HIV.^[9]^ Hepatitis C co-infection: • Co-infection with hepatitis C virus (HCV) is associated with an increased risk of cognitive impairment. The mechanisms underlying this risk are not fully understood but may involve shared neuroinflammatory pathways.^[10]^ Neurological opportunistic infections: • Opportunistic infections that affect the central nervous system, such as toxoplasmosis or cryptococcal meningitis, can contribute to cognitive impairment in individuals with HIV.^[11]^ Psychosocial factors: • Psychosocial factors, including stress, depression, and social isolation, can impact cognitive function. Depression, in particular, is common among individuals with HAND and can contribute to cognitive deficits.^[12]^ Genetic factors: • Certain genetic factors may increase susceptibility to HAND. Variations in genes related to neuroinflammation and synaptic function have been investigated as potential risk factors.^[13]^ Neurological biomarkers: • Elevated levels of neuroinflammatory biomarkers in the cerebrospinal fluid, such as neopterin and quinolinic acid, are associated with an increased risk of HAND.^[16]^ Immune activation: • Persistent immune activation and systemic inflammation, even in individuals on successful ART, are linked to an increased risk of cognitive impairment.^[29]^

It important to note that HAND is a complex condition influenced by these risk factors. Not all individuals with HIV will develop cognitive impairment, and the severity of HAND can vary widely. Early diagnosis, appropriate treatment, and management of these risk factors are crucial in reducing the risk and mitigating the impact of HAND.

## 4. Conclusion

HAND pose significant challenges to individuals living with HIV, affecting their cognitive functioning, mental health, and overall well-being. The diagnosis and management of HAND require a comprehensive approach that integrates medical, psychological, and social interventions.

The epidemiology of HAND demonstrates a high prevalence of cognitive impairment among people living with HIV, with varying degrees of severity and presentation patterns. Pathophysiologically, HAND is characterized by chronic inflammation, viral replication within the CNS, and neuronal damage.

The diagnosis of HAND involves a comprehensive assessment of cognitive functioning using neuropsychological tests, along with consideration of medical history and exclusion of other potential causes. Early detection and accurate diagnosis are crucial for implementing appropriate interventions and preventing further cognitive decline.

HAND management focuses on a multidisciplinary approach, including optimizing ART, cognitive rehabilitation, psychosocial support, and addressing comorbid mental health conditions. ART plays a crucial role in reducing viral load and inflammation, which can contribute to the preservation of cognitive function.

Mental health implications associated with HAND include depression, anxiety, social isolation, impaired daily functioning, reduced quality of life, treatment adherence challenges, and caregiver burden. These mental health aspects should be addressed through targeted interventions, including psychological counseling, support groups, and caregiver support programs.

Overall, research efforts should continue to advance our understanding of HAND and its underlying mechanisms and explore innovative therapeutic approaches to enhance cognitive function and improve mental health outcomes. Early detection, prompt management, and ongoing HAND monitoring can significantly improve the quality of life and overall well-being of individuals with HIV.

## Author contributions

**Conceptualization:** Chukwuka Elendu, Chinelo V. Okeke.

**Data curation:** Chukwuka Elendu, Jesse C. Peterson.

**Formal analysis:** Chukwuka Elendu.

**Investigation:** Chukwuka Elendu.

**Methodology:** Chukwuka Elendu, Chinonso B. Okoro.

**Project administration:** Chukwuka Elendu.

**Resources:** Chukwuka Elendu.

**Software:** Chukwuka Elendu.

**Supervision:** Chukwuka Elendu.

**Validation:** Chukwuka Elendu.

**Visualization:** Chukwuka Elendu.

**Writing – original draft:** Chukwuka Elendu.

**Writing – review & editing:** Chukwuka Elendu, Chinyere Mirian Aguocha, Chinelo V. Okeke.

## References

[1] Mitra P Sharman T. HIV Neurocognitive Disorders. [Updated 2022 Oct 20]. In: StatPearls. Treasure Island (FL): StatPearls Publishing; 2023. Available at: https://www.ncbi.nlm.nih.gov/books/NBK555954/32310414

[2] Thames AD Kuhn TP Mahmood Z. Effects of social adversity and HIV on subcortical shape and neurocognitive function. Brain Imaging Behav. 2018;12:96–108. 28130744 10.1007/s11682-017-9676-0 PMC5529267

[3] Moulignier A Costagliola D. Metabolic syndrome and cardiovascular disease impacts on the pathophysiology and phenotype of HIV-associated neurocognitive disorders. Curr Top Behav Neurosci. 2021;50:367–99. 31989463 10.1007/7854_2019_123

[4] Saylor D Dickens AM Sacktor N. HIV-associated neurocognitive disorder--pathogenesis and prospects for treatment. Nat Rev Neurol. 2016;12:234–48. 26965674 10.1038/nrneurol.2016.27 PMC4937456

[5] Su T Schouten J Geurtsen GJ. Multivariate normative comparison, a novel method for more reliably detecting cognitive impairment in HIV infection. AIDS. 2015;29:547–57. 25587908 10.1097/QAD.0000000000000573

[6] Cysique LA Brew BJ. Prevalence of non-confounded HIV-associated neurocognitive impairment in the context of plasma HIV RNA suppression. J Neurovirol. 2011;17:176–83. 21416169 10.1007/s13365-011-0021-x

[7] Gisslén M Price RW Nilsson S. The definition of HIV-associated neurocognitive disorders: are we overestimating the real prevalence? BMC Infect Dis. 2011;11:356. 22204557 10.1186/1471-2334-11-356 PMC3260107

[8] Ellis RJ Badiee J Vaida F. CD4 nadir is a predictor of HIV neurocognitive impairment in the era of combination antiretroviral therapy. AIDS. 2011;25:1747–51. 21750419 10.1097/QAD.0b013e32834a40cd PMC3867631

[9] Ojagbemi A. HIV Associated Neurocognitive Disorders Subsidence Through Citalopram Addition in Anti-retroviral Therapy (HANDS-CARE): a concept note. Front Neurol. 2021;12:658705. 34381409 10.3389/fneur.2021.658705 PMC8350562

[10] Kolson DL. Developments in Neuroprotection for HIV-Associated Neurocognitive Disorders (HAND). Curr HIV/AIDS Rep. 2022;19:344–57. 35867211 10.1007/s11904-022-00612-2 PMC9305687

[11] Lin SP Calcagno A Letendre SL. Clinical treatment options and randomized clinical trials for neurocognitive complications of HIV infection: combination antiretroviral therapy, central nervous system penetration effectiveness, and adjuvants. Curr Top Behav Neurosci. 2021;50:517–45. 33604875 10.1007/7854_2020_186 PMC8842834

[12] Force G Ghout I Ropers J. Improvement of HIV-associated neurocognitive disorders after antiretroviral therapy intensification: the Neuro+3 study. J Antimicrob Chemother. 2021;76:743–52. 33179033 10.1093/jac/dkaa473

[13] Eaton P Lewis T Kellett-Wright J. Risk factors for symptomatic HIV-associated neurocognitive disorder in adults aged 50 and over attending a HIV clinic in Tanzania. Int J Geriatr Psychiatry. 2020;35:1198–208. 32497330 10.1002/gps.5357

[14] Spooner R Ranasinghe S Urasa S. HIV-Associated neurocognitive disorders: the first longitudinal follow-up of a cART-treated cohort of older people in Sub-Saharan Africa. J Acquir Immune Defic Syndr. 2022;90:214–22. 35125473 10.1097/QAI.0000000000002934

[15] Flatt A Gentry T Kellett-Wright J. Prevalence and 1-year incidence of HIV-associated neurocognitive disorder (HAND) in adults aged ≥50 years attending standard HIV clinical care in Kilimanjaro, Tanzania. Int Psychogeriatr. 2023;35:339–50. 33757616 10.1017/S1041610221000156

[16] Sarfo FS Kyem G Asibey SO. Contemporary trends in HIV-associated neurocognitive disorders in Ghana. Clin Neurol Neurosurg. 2021;210:107003. 34715557 10.1016/j.clineuro.2021.107003 PMC8608734

[17] Kellett-Wright J Flatt A Eaton P. Screening for HIV-associated neurocognitive disorder (HAND) in adults aged 50 and over attending a government HIV clinic in Kilimanjaro, Tanzania comparison of the international HIV dementia scale (IHDS) and IDEA six item dementia screen. AIDS Behav. 2021;25:542–53. 32875460 10.1007/s10461-020-02998-9 PMC7846532

[18] Robbins RN Scott TM Gouse H. Screening for HIV-Associated neurocognitive disorders: sensitivity and specificity. Curr Top Behav Neurosci. 2021;50:429–78. 32677005 10.1007/7854_2019_117

[19] Trunfio M De Francesco D Vai D. Screening accuracy of mini Addenbrooke’s cognitive examination test for HIV-Associated neurocognitive disorders in people ageing with HIV. AIDS Behav. 2022;26:2203–11. 34982319 10.1007/s10461-021-03563-8

[20] Rappaport J Volsky DJ. Role of the macrophage in HIV-associated neurocognitive disorders and other comorbidities in patients on effective antiretroviral treatment. J Neurovirol. 2015;21:235–41. 25933548 10.1007/s13365-015-0346-y PMC4445403

[21] Lipton SA. HIV-related neuronal injury Potential therapeutic intervention with calcium channel antagonists and NMDA antagonists. Mol Neurobiol. 1994;8:181–96. 7999315 10.1007/BF02780669

[22] Banks WA Robinson SM Nath A. Permeability of the blood-brain barrier to HIV-1 Tat. Exp Neurol. 2005;193:218–27. 15817280 10.1016/j.expneurol.2004.11.019

[23] Valcour VG Ananworanich J Agsalda M. HIV DNA reservoir increases risk for cognitive disorders in cART-naïve patients. PLoS One. 2013;8:e70164. Erratum in: PLoS One. 2014;9(1). doi:10.1371/annotation/18c308ec-1cff-4e93-b4bb-6c9fc3a25a15. 23936155 10.1371/journal.pone.0070164 PMC3729685

[24] Weiss SL Selak MA Tuluc F. Mitochondrial dysfunction in peripheral blood mononuclear cells in pediatric septic shock. Pediatr Crit Care Med. 2015;16:e4–e12. 25251517 10.1097/PCC.0000000000000277 PMC4286436

[25] Kovalevich J Langford D. Neuronal toxicity in HIV CNS disease. Future Virol. 2012;7:687–98. 23616788 10.2217/fvl.12.57 PMC3632417

[26] Kallianpur AR Levine AJ. Host genetic factors predisposing to HIV-associated neurocognitive disorder. Curr HIV/AIDS Rep. 2014;11:336–52. 24996618 10.1007/s11904-014-0222-z PMC4121535

[27] Valcour V Shikuma C Shiramizu B. Higher frequency of dementia in older HIV-1 individuals: the Hawaii Aging with HIV-1 Cohort. Neurology. 2004;63:822–7. 15365130 10.1212/01.wnl.0000134665.58343.8d PMC1382180

[28] Mind Exchange Working Group. Assessment, diagnosis, and treatment of HIV-associated neurocognitive disorder: a consensus report of the mind exchange program. Clin Infect Dis. 2013;56:1004–17. 23175555 10.1093/cid/cis975 PMC3657494

[29] Robertson KR Smurzynski M Parsons TD. The prevalence and incidence of neurocognitive impairment in the HAART era. AIDS. 2007;21:1915–21. 17721099 10.1097/QAD.0b013e32828e4e27

[30] Farhadian S Patel P Spudich S. Neurological complications of HIV infection. Curr Infect Dis Rep. 2017;19:50. 29164407 10.1007/s11908-017-0606-5 PMC7456329

[31] Zhuang Y Qiu X Wang L. Combination antiretroviral therapy improves cognitive performance and functional connectivity in treatment-naïve HIV-infected individuals. J Neurovirol. 2017;23:704–12. 28791662 10.1007/s13365-017-0553-9 PMC5655604

[32] Heaton RK Clifford DB Franklin DR Jr. HIV-associated neurocognitive disorders persist in the era of potent antiretroviral therapy: CHARTER study. Neurology. 2010;75:2087–96. 21135382 10.1212/WNL.0b013e318200d727 PMC2995535

[33] Clifford DB. HIV-associated neurocognitive disorder. Curr Opin Infect Dis. 2017;30:117–22. 27798498 10.1097/QCO.0000000000000328 PMC5382956

[34] Rao VR Ruiz AP Prasad VR. Viral and cellular factors underlying neuropathogenesis in HIV associated neurocognitive disorders (HAND). AIDS Res Ther. 2014;11:13. 24894206 10.1186/1742-6405-11-13 PMC4043700

[35] Brew BJ Crowe SM Landay A. Neurodegeneration and ageing in the HAART era. J Neuroimmune Pharmacol. 2009;4:163–74. 19067177 10.1007/s11481-008-9143-1

[36] Robbins RN Joska JA Thomas KG. Exploring the utility of the Montreal Cognitive Assessment to detect HIV-associated neurocognitive disorder: the challenge and need for culturally valid screening tests in South Africa. Clin Neuropsychol. 2013;27:437–54. 23336183 10.1080/13854046.2012.759627 PMC3631431

[37] Letendre SL Mills AM Tashima KT. ING116070: a study of the pharmacokinetics and antiviral activity of dolutegravir in cerebrospinal fluid in HIV-1-infected, antiretroviral therapy-naive subjects. Clin Infect Dis. 2014;59:1032–7. 24944232 10.1093/cid/ciu477 PMC4166983

[38] Schouten J Cinque P Gisslen M. HIV-1 infection and cognitive impairment in the cART era: a review. AIDS. 2011;25:561–75. 21160410 10.1097/QAD.0b013e3283437f9a

[39] Phillips AN Neaton J Lundgren JD. The role of HIV in serious diseases other than AIDS. AIDS. 2008;22:2409–18. 19005264 10.1097/QAD.0b013e3283174636 PMC2679976

[40] Thames AD Kim MS Becker BW. Medication and finance management among HIV-infected adults: the impact of age and cognition. J Clin Exp Neuropsychol. 2011;33:200–9. 20694873 10.1080/13803395.2010.499357 PMC3616485

[41] Ettenhofer ML Foley J Castellon SA. Reciprocal prediction of medication adherence and neurocognition in HIV/AIDS. Neurology. 2010;74:1217–22. 20220123 10.1212/WNL.0b013e3181d8c1ca PMC2865732

[42] Heaton RK Marcotte TD Mindt MR. The impact of HIV-associated neuropsychological impairment on everyday functioning. J Int Neuropsychol Soc. 2004;10:317–31. 15147590 10.1017/S1355617704102130

[43] Sacktor N Nakasujja N Redd AD. HIV subtype is not associated with dementia among individuals with moderate and advanced immunosuppression in Kampala, Uganda. Metab Brain Dis. 2014;29:261–8. 24515303 10.1007/s11011-014-9498-3 PMC4024330

[44] Korten V Ay U Hari E. Prevalence of HIV-associated neurocognitive disorder (HAND) in Turkey and assessment of Addenbrooke’s Cognitive Examination Revised (ACE-R) test as a screening tool. HIV Med. 2021;22:60–6. 32964651 10.1111/hiv.12957

